# Potential carbon loss associated with post-settlement wetland conversion in southern Ontario, Canada

**DOI:** 10.1186/s13021-018-0094-4

**Published:** 2018-04-20

**Authors:** Eunji Byun, Sarah A. Finkelstein, Sharon A. Cowling, Pascal Badiou

**Affiliations:** 1 Department of Earth Sciences, University of Toronto, 22 Russell Street, Toronto, ON M5S 3L1 Canada; 2 Institute for Wetland and Waterfowl Research, Ducks Unlimited Canada, PO Box 1160, Stonewall, MB R0C 2Z0 Canada

**Keywords:** Wetland, Carbon sink, Peat, Holocene carbon cycle, Wetland conversion, Land use change

## Abstract

**Background:**

Natural wetlands can mitigate ongoing increases in atmospheric carbon by storing any net balance of organic carbon (peat) between plant production (carbon uptake) and microbial decomposition (carbon release). Efforts are ongoing to quantify peat carbon stored in global wetlands, with considerable focus given to boreal/subarctic peatlands and tropical peat swamps. Many wetlands in temperate latitudes have been transformed to anthropogenic landscapes, making it difficult to investigate their natural/historic carbon balance. The remaining temperate swamps and marshes are often treated as mineral soil wetlands and assumed to not accumulate peat. Southern Ontario in the Laurentian Great Lakes drainage basin was formerly a wetland-rich region that has undergone significant land use change since European settlement.

**Results:**

This study uses southern Ontario as a case study to assess the degree to which temperate regions could have stored substantial carbon if it had not been for widespread anthropogenic land cover change. Here, we reconstruct the full extent and distribution of natural wetlands using two wetland maps, one for pre-settlement conditions (prior to 1850 CE) and the other for modern-day patterns of land use (2011 CE). We found that the pre-settlement wetland cover decreased by about 56% with the loss most significant for marshes as only 11% of predicted pre-settlement marshland area remains today. We estimate that pre-settlement wetlands held up to ~ 3.3 Pg of carbon relative to ~ 1.3 Pg for present-day (total across all wetland classes).

**Conclusions:**

By not considering the recent carbon loss of temperate wetlands, we may be underestimating the wetland carbon sink in the pre-industrial carbon cycle. Future work is needed to better track the conversion of natural wetlands globally and the associated carbon stock change.

**Electronic supplementary material:**

The online version of this article (10.1186/s13021-018-0094-4) contains supplementary material, which is available to authorized users.

## Background

Natural wetlands can act as a long-term terrestrial carbon sink by storing peat (plant organic litter undergoing very slow decomposition) under waterlogged conditions [1]. Also, as an active methane (CH_4_) source, the past expansion of global wetlands has been linked to Holocene carbon dioxide (CO_2_) and CH_4_ fluctuations [2, 3]. However, poor quantification of paleo-wetland extent and carbon balance limits robust coupling [4]. Significant wetland conversion by human land use in the heavily populated temperate zone started earlier than the industrial era in many regions and must have reduced the natural wetland carbon storage since that time [5, 6]. The implications of anthropogenic wetland conversion on carbon stocks need to be more explicitly quantified at the global scale, but regional studies are a critical first step (e.g., [7–10]).

The majority of the global peatland carbon pool is contained within northern boreal and sub-arctic regions located poleward of 45°N. In the form of bogs and fens, these regional wetlands are usually characterized by thick and extensive peat deposits, either moss-covered or sparsely vegetated with shrubs and herbaceous plants. With increasing concerns about future climate change and the fate of such large organic carbon pools in climatically sensitive areas, efforts to improve knowledge about global peatlands have centered upon northern boreal/subarctic regions ([11] and ref. therein). The perceived importance of northern peatlands has resulted in less emphasis on to other wetland types of mid-latitude regions, such as swamps and treed-fens, which containing larger and more abundant trees compared to the northern open fen classification (e.g., [12]). While the environmental conditions associated with tree growth (e.g., improved soil aeration and nutrient-rich water supply) seem less optimal in terms of long-term preservation of organic matter, some recent studies suggest comparable peat accumulation and high organic carbon densities of the tree-covered temperate wetlands [12–14].

**Fig. 1:**
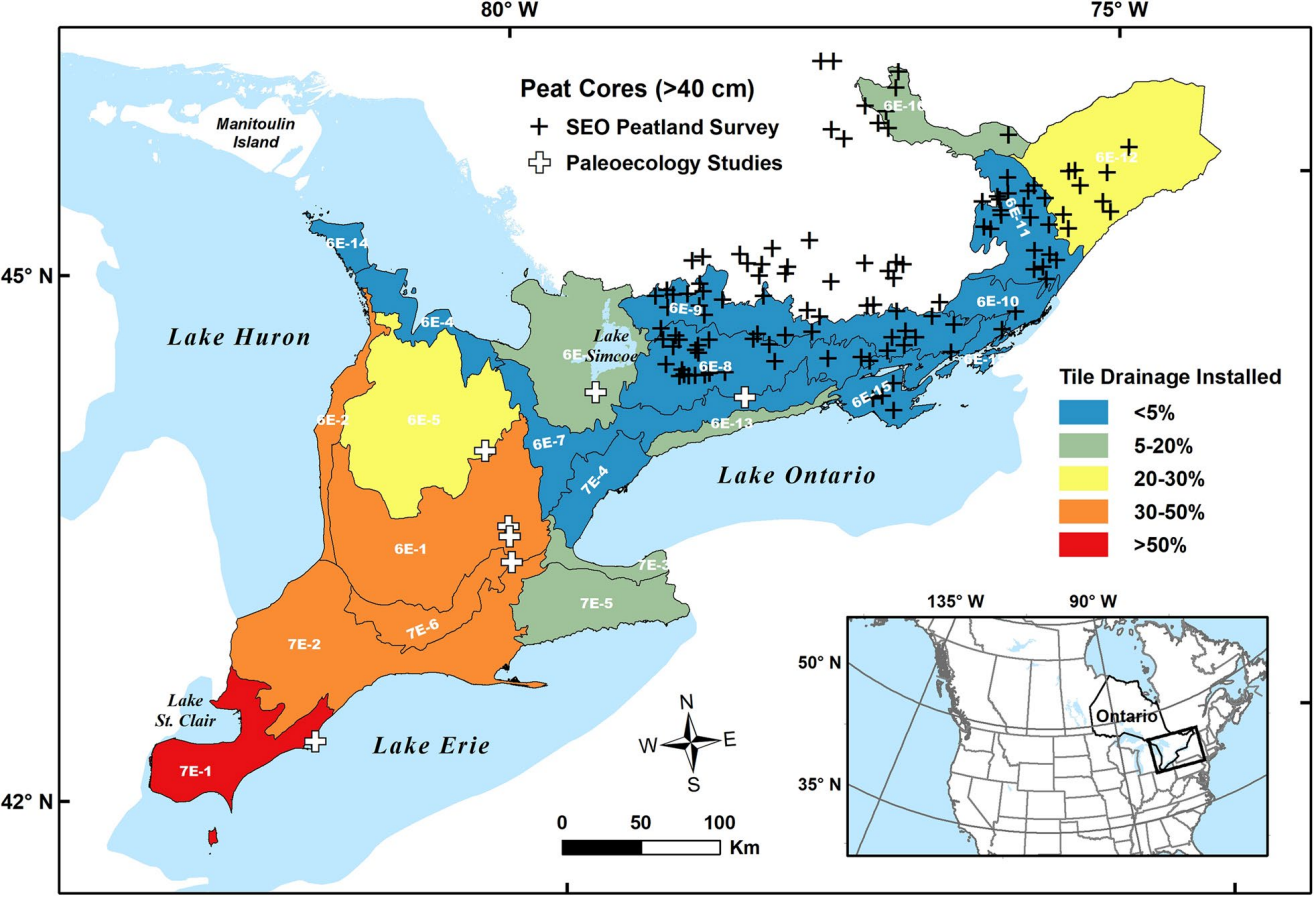
Map of southern Ontario study area including 21 Ecodistricts from 6E−1 to 16 and 7E−1 to 6 (excluding 6E−3) [25]. Shading represents percentage of area where tile drainage system has been installed, as a proxy for the degree of anthropogenic land use change associated with wetland conversion. Black crosses indicate locations for peat cores data from the southeastern Ontario (SEO) peatland survey [24] and white crosses indicate wetland sediment cores data obtained from paleoecological studies [17–23]. All the marked location has the average peat depth exceeding 40 cm. See Additional file 1: Figure S2 for an example of the original SEO peatland survey map

Since many wetlands were drained before inventories of carbon pools took place, studies are limited by uncertain pre-disturbance extent and lack of carbon density measurements for pristine conditions as indicated by Bridgham et al. [7]. Southern Ontario (Canada), particularly the southwestern portion, is notable for its dramatic loss of natural wetlands since European settlement [15, 16]. Some wetlands survived and have been the focus of paleoenvironmental studies, mostly with peat cores containing Holocene-age basal sediments [17–23]. Analyses of these wetland cores indicate considerable peat thicknesses and organic matter densities (see Fig. 1 for locations), however there has never been an effort to systematically quantify net carbon uptake. In the 1980s, the still relatively abundant wetlands in southeastern Ontario (SEO) were investigated for potential peat extraction and resource development [24]. The SEO peatland survey reported significant peat accumulation from many regional swamp and freshwater marsh sites, prompting a reconsideration of these systems, not as overall mineral-soil wetlands (e.g., [6, 7]), but as part of northern peatlands. Studies compiling data from soil cores suggest that various wetland sites other than bogs and fens are also important in wetland organic carbon stock accounting [9, 10].

In this study, we aim to reassess southern Ontario wetlands as an underestimated middle-latitude peat and organic carbon stock. Maps of pre-settlement large (> 10 ha) wetlands and current detailed wetland distributions were combined to reconstruct the extent and distribution of wetlands in five categories (tree swamp, shrub swamp, marsh, fen, and bog) before anthropogenic land use conversion since 1850 CE. The resulting natural wetland cover represents the available potential for Holocene carbon storage, as it integrates carbon density data for the different wetland classes from the SEO peat resource survey [24]. Decreased natural wetland cover corresponds to anthropogenic land carbon emission over the settlement period. This study estimates wetland carbon loss by first categorizing current land uses for historic wetlands. By doing so, we show that the treed wetlands (swamps) and marshes which were so prevalent in the study region prior to 1850 CE, have the potential to store large amounts of organic carbon, wetland types that have been overlooked in analyses of Holocene peatland carbon stock.

## Methods

### Study area

We focus our study on the terrain falling south of the Precambrian shield in Ontario, Canada (Fig. 1). Based on the ecological land classification (ELC) of Ontario [25], our study area belongs to the Mixedwood Plains Ecozone. This Ecozone is composed of two Ecoregions (6E: Lake Simcoe-Rideau; 7E: Lake Erie-Lake Ontario), in turn sub-divided into Ecodistricts, of which we include 6E−1 to 6E−16 (excluding 6E−3 of Manitoulin Island) and 7E−1 to 7E−6 in our study. The climate is slightly different between 6E (annual mean temperature, 4.9–7.8 °C) and 7E (6.3–9.4 °C) but overall the mildest in Canada and humid due to proximity to the Great Lakes. Growing season length exceeds 200 days (up to ~ 240 days) and precipitation ranges from 720 to 1000 mm per year [25].

The Canadian Wetland Classification system classifies most part of southern Ontario as ‘Eastern Temperate Wetlands’ [26]. Dominant wetland types are hardwood swamp with maple trees (*Acer saccharinum*) and freshwater marsh with cattails (*Typha* spp.). Those wetlands were naturally established as a part of the post-glacial landscape, but drainage and conversion to croplands have been widespread since European settlement [26]. This study contrasts two periods of natural wetlands: one before European settlement (‘pre-settlement wetlands’ in 1800 CE) and the other with modern-day land use (surveyed in 2011).

### Map overlay analysis

In the absence of historical wetland maps for pre-disturbance conditions, soil maps can be used as an alternative as wet soils are proxies for wetland locations. This was the approach used by Snell [15] (Additional file 1: Figure S1 for details). Ducks Unlimited Canada (DUC) [16], building upon the work of Snell [15], digitized the soil maps using GIS software, and added topographic and hydrological data to better approximate the potential distribution of wetlands in the pre-settlement period.

Wetland conversion was identified by areas where the pre-settlement wetlands overlap with non-wetland areas in the modern day (2011 CE) land use map (Additional file 1: Figure S1). In Snell’s work, maps for 1967 and 1982 land uses were superimposed on the soil maps, and later the digitized analyses of DUC [16] also included recent trends between 1982 and 2002 using the provincial land cover information system [Southern Ontario Land Resource Information System (SOLRIS) Version 1.0]. In our study, we utilize the most recent version of SOLRIS (V2.0) for the year 2011; the modern-day (2011) wetland map was obtained by extracting wetland polygons in five classes: tree swamp, shrub swamp, marsh, bog, and fen (Additional file 1: Table S1).

Because obtaining the maximum potential area of Holocene natural (pre-settlement) wetlands and carbon storage was our objective, the 2011 wetland map was used to adjust the DUC’s pre-settlement map. By overlaying the 2011 wetland layer (SOLRIS V2.0) to the DUC’s map layer, we assigned the five wetland classes of SOLRIS V2.0 on the pre-settlement wetland polygons for more explicit carbon stock estimation by wetland class. Each pre-settlement polygon had spatial proximity with type-classified polygons from the 2011 wetland map, and the spatially closest one was allocated to the same wetland class. Central to this methodology is the assumption that the current wetlands are fragments of the larger past wetland. Our pre-settlement layer also included small wetlands (< 10 ha), which were excluded from the previous report [16]. These smaller wetlands were excluded from the DUC report due to the coarser map scale used in the data of Snell [15]. The smallest wetland in our pre-settlement map was 0.5 ha for the polygons from the SOLRIS layer (the minimum mapping unit in the source data), and 1 ha from the DUC layer (determined to avoid slivered polygons; larger features assumed to be wetland features by the original map overlay analysis by DUC). Overall, wetlands found in the current land cover but not in the pre-settlement layer (summing up to ~ 4656 km^2^ in extent), mostly from those small wetlands, were added to our pre-settlement wetland map. It was unlikely that this additional area (~ 4656 km^2^) was caused by creation of new wetlands since the onset of the settlement period and land use expansion.

By deleting the areas recognized as modern-day wetlands from the full pre-settlement cover, the map layer for ‘converted wetlands’ was created. Then, the converted wetland layer was overlaid onto the SOLRIS V2.0 (2011) land cover layer to display the wetland conversion. Different land uses were summarized by each ecodistrict and by our five wetland classes.

### Wetland carbon stock estimation

The pre-settlement wetland map represents the maximum capacity of natural carbon held by wetlands for the Holocene by assuming the cumulative increase since the deglaciation (e.g., [2, 3, 11]), and modern-day map is comparable to how much has been altered by human land use change (e.g., pre-settlement wetland converted into farmland). Wetland carbon stocks were estimated for both pre-settlement and post-settlement (modern-day) periods using the cumulative carbon mass data obtained from the SEO peat survey (using peat depth, bulk density, and carbon content; see Additional file 1: Appendix S1 for detailed procedures).

## Results

### Pre-settlement, converted, and current wetlands

**Fig. 2:**
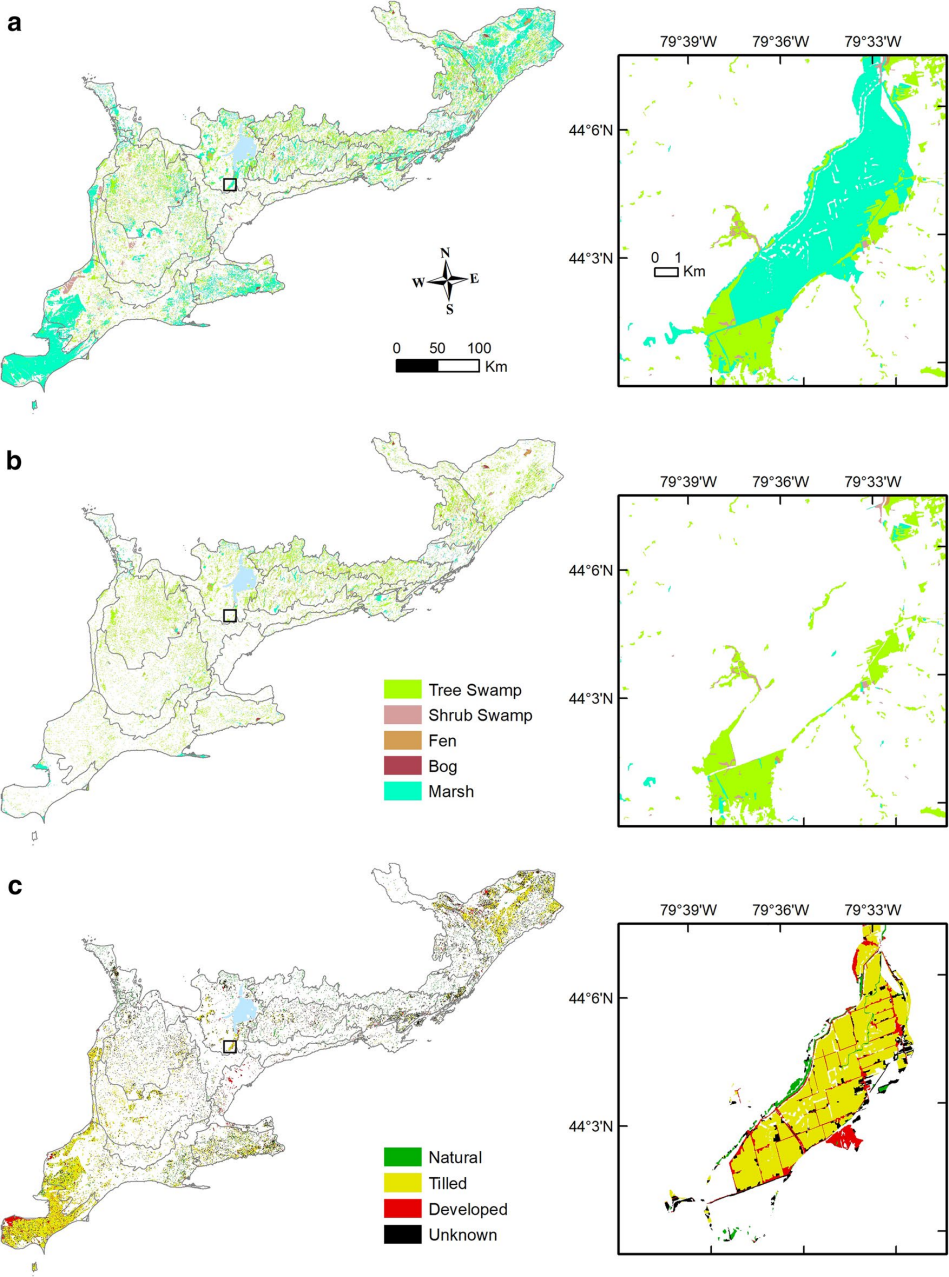
Map overlay results showing **a** distribution of pre-settlement wetlands colour coded by wetland class, **b** current wetlands colour coded by wetland class, and **c** current land cover classification for converted wetlands, coloured by land use type. For wetlands (**a**, **b**) based on the wetland classification used in SOLRIS and the SEO peat survey (Additional file 1: Table S1; Appendix S1) and the converted land cover types (**c**) from Southern Ontario Land Resource Information System Version 2.0 (SOLRIS V2.0) and Table 1 [27]. Insets show the Holland Marsh, a large pre-settlement marsh and treed swamp complex. High resolution figures are available in the digital version

From the map overlay analysis, three wetland maps were created for southern Ontario (Fig. 2) with classification into five wetland classes (Additional file 1: Table S1 for detailed information). The total area of the study region is 83,810 km^2^ (Fig. 1). Of this total, the extent of pre-settlement wetlands was estimated at ~ 24,984 km^2^ (~ 30% of total land cover), reflecting the sum of the current wetlands (~ 11,032 km^2^) and converted wetlands (~ 13,953 km^2^) as in Table 1. Among the five wetland types, marsh is the largest portion of the pre-settlement cover (Table 1 and Fig. 2a), most notably the extensive marshes of southwestern Ontario (currently agricultural lands, Fig. 2c), and the extant marshes south of Lake St. Clair (still existing as marsh, Fig. 2b). Tree swamp is now the most frequent wetland class (Fig. 2b) and occupies 75.6% of the total current wetlands by areal extent, compared to 40.5% of the total historical wetland extent in Table 1.

**Table 1:** Wetland conversion summary from map overlay analysis

	Tree swamp	Shrub swamp	Fen	Bog	Marsh	Total
Pre-settlement extent (km^2^)	10,111.68	2191.73	54.75	108.90	12,517.24	24,984.30
Current (2011 CE) extent (km^2^)	8341.62	1167.46	53.07	88.92	1380.57	11,031.64
Converted extent (km^2^)	1770.06	1024.27	1.68	19.98	11,136.67	13,952.66
% of Pre-settlement						
Remaining (2011 CE)	82.5%	53.3%	96.9%	81.7%	11.1%	44.2%
Converted since 1800 CE	17.5%	46.7%	3.1%	18.3%	88.9%	55.8%
% of converted pre-settlement						
Cultivated (tilled^a^, tree planting)	50.8%	50.4%	29.2%	42.9%	58.6%	57.0%
Developed (building, road, extraction)	8.7%	9.5%	4.4%	5.3%	6.9%	7.3%
Natural^b^ (total)	12.4%	11.6%	45.2%	5.0%	9.7%	10.2%
(Rocky—beach, cliff, alvar)^c^	(0.17%)	(0.17%)	–	–	(0.80%)	(0.65%)
(Grassland—prairie, tallgrass)	(0.04%)	(0.83%)	–	–	(0.34%)	(0.34%)
(Forest—conifer, mixed, deciduous)	(92.9%)	(85.9%)	(95.7%)	(85.3%)	(80.6%)	(83.0%)
(Open water—deep, unvegetated)	(6.9%)	(13.1%)	(4.3%)	(14.7%)	(18.2%)	(16.0%)
Undifferentiated^d^	28.1%	28.6%	21.1%	46.7%	24.8%	25.5%

^a^ Managed agricultural fields for annual crops (i.e., ‘tilled’) account for > 95% of the cultivated pre-settlement wetlands; tree plantation is relatively sparse

^b^ This category can include possible errors from the current wetland mapping or an actual ecosystem change resulting from the lowering of water tables in response to drainage in the surrounding area (see main text)

^c^ This class was exempted from peat carbon stock calculations in Table 4 (see Additional file 1: Appendix S1 for more details)

^d^ Areas were unidentifiable as specific land classes and may include pastures, orchards, vineyards, abandoned farmlands, urban brownfields, the edge of transportation corridors, upland thicket, and unclassified wetlands [27]

**Fig. 3:**
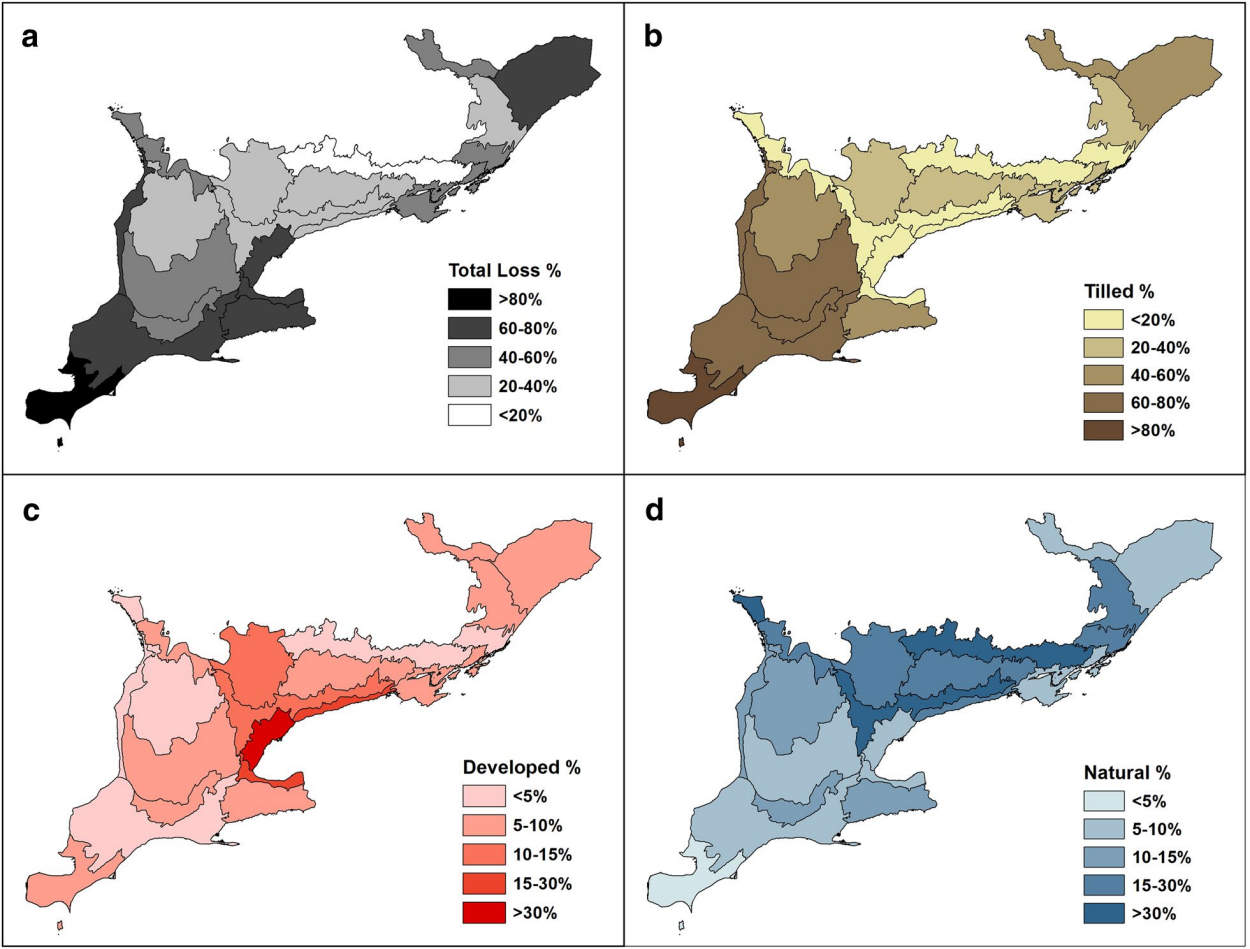
Wetland conversion by Ecodistrict: **a** total loss (%) for current land type of converted wetlands (in % cover) including **b** tilled (agricultural land), **c** developed (urbanized), and **d** natural land cover (non-agricultural and non-urban land classes including forests; Table 1)

Agricultural land use is the most common driver of the pre-settlement wetland conversion regardless of the wetland type (Table 1), but there are some local exceptions. In the densely populated areas along the coast of Lake Ontario (7E−3 and 7E−4 in Fig. 1), conversion of natural wetlands is due mostly to urban development rather than agriculture (Fig. 3c). Some ecodistricts with relatively small wetland loss (e.g., 6E−9 at the boundary of the Precambrian Shield; Fig. 1) have a higher proportion of transition to other natural land types (Fig. 3d) mostly attributable to afforestation (Table 1). The wetland-to-forest conversion may include the current swamp not recognized by SOLRIS mapping and possibly the past wetlands where lowering of water levels promoted increasing tree cover (see Additional file 1: Table S1 for tree swamp classification).

### Southern Ontario wetlands carbon storage

The parameters for wetland carbon stock calculations were obtained from the SEO peatland survey and presented in Tables 2 and 3 (see Additional file 1: Appendix S1 for data acquisition and calculation). The average peat depths by different wetland types (Table 2) were applied to convert the organic carbon density (Table 3) to the cumulative carbon mass for each wetland type (Table 4).

**Table 2:** Estimated peat depths for SEO peatlands

	Southeastern Ontario peatlands survey			Estimated peat depth (m), mean [SD]
	Peat depths recorded sites (cores per site)^a^	Total peat cores	Peat depth range^b^ (m)	
Conifer swamp	23 (4–140)	556	0.7–5.0	2.2 [1.1]
Mixed swamp	25 (3–148)	799	0.2–3.7	1.7 [0.8]
Deciduous swamp	29 (3–161)	663	0.4–4.3	1.3 [1.0]
Shrub swamp	32 (3–91)	415	0.3–6.0	1.7 [1.3]
Fen	20 (2–37)	180	0.9–4.4	2.4 [1.0]
Bog	13 (2–36)	159	1.9–3.9	2.9 [0.8]
Marsh	19 (2–40)	138	0.4–3.5	1.5 [0.9]
(All sites)	161 (2–161)	2910	0.2–6.0	1.9 [1.2]

^a^ Peatland sites with only one peat core sampled were excluded from the estimation because those depths could be biased and likely represent the deepest center of peatland sites [24]

^b^ Average depth of peat cores from a site; the depth of each peat core was not reported in the SEO peatland survey report unless there was only one core from the site

**Table 3:** Estimated peat organic carbon density for each SEO peatland class

	Southeastern Ontario peatlands survey				Estimated peat organic carbon density^c^ (kg C m^−3^), mean [SD]
	Analyzed peat samples (peat cores)^a^	Sample depth range (cm)	Peat dry bulk density (g cm^−3^), mean [SD]	Ash (%)^b^, mean [SD]	
Conifer swamp	85 (15)	0–450	0.19 [0.06]	10.0 [4.3]	81.6 [24.2]
Mixed swamp	49 (13)	0–730	0.14 [0.05]	8.5 [4.4]	59.3 [19.4]
Deciduous swamp	28 (8)	0–400	0.18 [0.05]	12.4 [4.9]	72.5 [18.4]
Shrub swamp	36 (10)	0–320	0.19 [0.07]	9.9 [4.7]	79.7 [30.5]
Fen	38 (11)	0–370	0.16 [0.04]	9.9 [4.4]	66.5 [15.7]
Bog	42 (9)	0–640	0.13 [0.05]	4.3 [3.8]	59.7 [22.5]
Marsh	9 (2)	0–425	0.22 [0.04]	9.6 [3.4]	94.5 [18.4]
(All samples)	287 (68)	0–730	0.17 [0.06]	9.1 [4.9]	71.9 [24.5]

^a^ One to seven peat samples were analyzed from one peat core section at irregular intervals from top to bottom [24, 28]

^b^ Inorganic composition of each peat sample determined from loss on ignition at 750 °C for > 1 h [28]

^c^ See Additional file 1: Appendix S1 for a detailed derivation

**Table 4:** Estimated cumulative carbon mass and southern Ontario wetland carbon stocks

	Estimated cumulative carbon mass^a^ (kg C m^−2^), mean [SD]	Areal extent estimate (km^2^)		Carbon stock (Tg C), estimate [SD]^c^	
		Pre-settlement wetlands^b^	Current wetlands	Pre-settlement wetlands	Current wetlands
Tree swamp^d^		10,111	8342	1156 [494]	954 [408]
Coniferous	180 [104]				
Mixed	101 [58]				
Deciduous	94 [76]				
Shrub swamp	136 [116]	2192	1167	298 [254]	159 [135]
Fen	160 [76]	55	53	9 [4]	8 [4]
Bog	173 [81]	109	89	19 [9]	15 [7]
Marsh	142 [89]	12,509	1381	1776 [1118]	196 [123]
Total		24,975	11,032	3258 [1249]	1332 [447]
(All)^e^	137 [98]			3422 [2448]	1511 [1081]

^a^ Product of the estimated average peat depth (Table 2) and the organic carbon density (Table 3). See Additional file 1: Appendix S1 for more details

^b^ Some values are marginally smaller than the pre-settlement totals shown in Table 1, as natural area with shallow substrates (i.e., ‘rocky’—beach, cliff, alvar) have been subtracted. See Additional file 1: Appendix S1 for a detailed procedure

^c^ Product of the cumulative carbon mass and the wetland extent. Estimated uncertainties (SD) reflect only the carbon density estimates; the carbon stock values have greater uncertainties from the errors of wetland mapping which are not accounted for here (Additional file 1: Appendix S1)

^d^ Tree swamp extent was proportioned based on the current forest cover ratio (coniferous:mixed:deciduous = 22:25:54) of the study area, assuming a similar distribution of tree species in forests and forested wetlands

^e^ From the cumulative carbon mass estimate from all sites and samples (the bottom rows of Tables 2 and 3). The carbon stocks in the same row are derived from this average carbon mass, without classifying by wetland types

Carbon stock changes were calculated for southern Ontario wetlands for the two periods, the pre-settlement 3.3 ± 1.2 Pg C (1 SD) and the current 1.3 ± 0.4 Pg C (1 SD) resulting in the difference of 1.9 ± 1.3 Pg C (1 SD) as the possible carbon loss due to wetland conversion (Table 4). The carbon loss contribution by each wetland class was proportional to the extent of conversion, and hence most significant change in carbon storage is in the conversion of marshes that explain 82% of the estimated total. However, wetland classes vary in terms of carbon storage potential. In Table 4, for example, organic carbon mass per unit area of coniferous swamp (180 ± 104 kg C m^−2^, 1 SD) is estimated almost twice that of deciduous swamp (94 ± 76 kg C m^−2^, 1 SD). Thus, a critical step in obtaining accurate estimates of potential wetland carbon storage is the assignment to correct wetland classes, as each class has different carbon storage potential. In this study, if the wetlands were not categorized and the single average carbon mass ‘(All)’ multiplied to ‘Total’ wetland extent, the resulting carbon stock values and uncertainties (1 SD) are larger than the sum of classified estimates for both pre-settlement and current wetlands (Table 4).

## Discussion

### Reconstruction of pre-settlement wetlands

Our effort to include small wetlands (< 10 ha) in our pre-settlement reconstruction increased the total estimated extent of pre-settlement wetlands by 25% compared to previous work [15, 16]. Our methodology results in a lower estimate for percentage wetland loss; for example, 72% wetland loss was reported in DUC [16] versus our value of 55.8% in Table 1. Despite our attempt to include small wetlands (1–10 ha) that were excluded by previous studies [15, 16], we may still be underestimating the amount of ‘small’ wetlands present historically. Small wetlands are clearly important for estimating overall wetland extent [29]. If those small wetlands (< 1 ha) could be fully mapped for pre-settlement wetlands, the loss ratio might be higher than the estimated 55.8%.

In the pre-settlement reconstruction, the marsh wetland class is overwhelmingly dominant and swamp wetlands are proportionately much smaller; both observations contrast to modern-day (Fig. 2). In the absence of actual historical maps of wetland extent, some larger-scale marshes from the past can be determined from the region’s settlement history, such as the Holland Marsh (now croplands), Long Point (conservation and recreational use), and Toronto Harbour (infilling and railway construction) [30]. In our reconstruction (Fig. 2), the Holland Marsh (near Lake Simcoe) and Long Point (Lake Erie) areas remain as marshes. Also, the southeastern shoreline of Lake St. Clair was known for its extensive marsh cover prior to the intensive drainage and agricultural expansion since the late 1800s [31]. Following drainage and conversion, some small fragmented areas of marshland may have continued to exist in the region [26, 32], with a relatively extensive marsh remained along the lakeshore of Lake St. Clair (Fig. 2b). From the workload perspective of early European settlers, marshes would have been easier to convert to cropland than forested swamp landscapes simply due to the reduction in heavy woody plants. Indeed, the surrounding treed swamps of Holland Marsh farmlands (Fig. 2 insets) have remained almost untouched since their discovery (e.g., the northwestern ‘tamarack swamp’ in an early surveyor’s note in 1804; [30]).

The transition to the current swamp-dominated landscape may also be associated with natural wetland succession that happened in this region [18, 26, 33] and not with selective anthropogenic disturbance alone. The abundance of swamps may be self-perpetuating because once trees become dominant within the wetland, they can act to stabilize the system; disturbances by beavers notwithstanding [33–35]. The tendency for trees to eventually dominate a wetland landscape can be observed in SEO peat core data, where successional shifts are documented from sedge to woody peat through time (Additional file 1: Appendix S2 for further discussion).

Therefore, current dominance of swamps likely resulted from both natural wetland succession and anthropogenic disturbance. This change implies that the wetland conditions identified at the time of modern-day investigations do not necessarily reflect past wetland distribution nor the associated ability to sequester carbon. Due to the lack of information on historical conditions and peat accumulation, potential peat deposits and cumulative organic carbon stores should not be calculated based only on sparse observations of the more typically studied peatland types (e.g., open bogs and fens).

### Wetland conversion and carbon loss potential

Our results suggest that more than half of the natural wetlands have transformed to other land types, eventually forcing up to ~ 1.9 Pg C of organic carbon out of the original waterlogged systems (Table 4). This amount supports an underestimation of soil organic carbon (SOC) loss due to anthropogenic land use, especially from regions found to have had abundant wetlands in the past. Due to possibly much higher organic matter content in the form of peat and its vulnerability to degradation (induced by drainage, oxygenation, and compaction), cumulative carbon loss by direct use of wetlands (up to carbon mass values in Table 4 if the entire peat layer has been extracted) would far exceed the upper limit of worldwide SOC loss by human land use (54 Mg C ha^−1^ in 2-m depth; 95% confidence interval) in the prediction model of Sanderman et al. [36]. The ~ 1.9 Pg of carbon stock, only from our study region’s wetland loss, is comparable to top ten listed SOC losses by country, in which Canada is not included (in supplementary information of [36]).

Modernization of the current landscape is estimated to have occurred between 1835 CE to 1967 CE in southern Ontario [15]. Over this same period, fossil fuel emissions within Canada are estimated to be 2.15 Pg C [37]. This industrial carbon emission is estimated without considering past drainage of wetland systems, therefore the value may be significantly underestimated [5]. According to our results, the amount of land carbon released due to the settlement and wetland conversion in southern Ontario would almost double this national fossil carbon emission [37]. Other anthropogenic disturbances related to land-use change such as deforestation may have also resulted in a release of terrestrial carbon to the atmosphere (e.g., [38]).

However, the rate of carbon loss associated with wetland conversion will ultimately depend on the type of anthropogenic disturbance (i.e., gradual or abrupt). Drainage of wetlands (gradual) is far more important than peat extraction (abrupt) in southern Ontario because the peat was found to be mostly unsuitable for use as fuel or horticultural resources [24]. Instead, agricultural use of wetland surfaces (i.e., requiring drainage) was deemed a more productive and affordable land use alternative [32]. A very small proportion of the pre-settlement wetland removal is in fact attributable to direct peat extraction (1.8% of the total ‘Developed’ in Table 2).

Wetland drainage and row crop harvesting began during the settlement period, and are generally represented as ‘Tilled’ land use classification (tillage or tilling for soil management; cf. ‘Tile drainage’ in Fig. 1 is a common method to lower the water table prior to tillage or other land uses). Wetlands managed for farming gradually lose the surface peat by oxidation and compression, resulting in overall subsidence of the land surface. Soil cores from a historic fen in Switzerland, with similar agricultural management over ~ 140 years, recorded 16–49% loss of ‘pre-drainage’ organic carbon (‘Table 2’ of [39]). Although Leifeld et al. [39] caution against applying a single default value all the southern Ontario wetland-to-cropland area is assumed here to have experienced the same level of subsidence and peat oxidation. This approach was taken due to lack of detailed information on rates of subsidence or oxidation for our region. Thus, we applied a ross rate of 49%, as was done for the Staatswald site [39] based on the similar pre-drainage carbon mass of southern Ontario marshes (142 kg C m^−2^ as in Table 4) and the high subsidence rate (~ 3.3 cm year^−1^) recorded from the Holland Marsh site in Ontario, which remains one of the only sites where detailed subsidence rates are available for drained wetlands in the study region [40].

Accordingly, the ~ 1.9 Pg of estimated carbon loss can be partitioned into ‘completed’ (c) and ‘potential’ (p) loss. Proportional to the 2011 land classes of the converted wetlands (Table 1), 57.0% of the total disturbed wetland carbon is under agricultural land use (1.1 Pg C), 7.4% developed (0.14 Pg C), 10.2% natural (0.20 Pg C), and 25.5% unspecified lands (0.48 Pg C). Among these, the developed wetland is likely accompanied by peat extraction and completed the carbon loss (Dc: 0.14). If all the drained peats under the croplands have now reached the 49% carbon loss by subsidence, 0.54 Pg C has been released (Agriculture completed, Ac: 0.54), leaving 0.56 Pg C in soils either translocated downwards or mixed with mineral substrates (Agriculture potential, Ap: 0.56). The former wetlands now in the “natural cover” class (10.2%) most likely retain the wetland accumulated carbon relatively intact (Np: 0.20). The 0.48 Pg C under the undifferentiated lands is unknown with respect to carbon release, so here it is considered as ‘potential’ loss (Up: 0.48). In summary, among the total ~ 1.9 Pg C, only 0.7 Pg C (0.54_Ac_ + 0.14_Dc_) can be said to have been ‘completely’ lost, and 1.2 Pg C (0.56_Ap_ + 0.20_Np_ + 0.48_Up_) needs further investigation for possible on-going or future loss.

### Underestimated Holocene peat accumulation

A comprehensive database for Canadian peatlands (Tarnocai database hereafter) has been available since its first release date in 2005, with revisions made in 2011 [41]. The total peatland carbon stock in that database has been used in synthesis studies of northern peatlands [3, 11]. We compare our results to the information given for southern Ontario in the Tarnocai database. We argue that all wetland classes have the potential to store considerable carbon in the form of peat, so our carbon values represent the upper range of wetland peat carbon stock for the region. According to the Tarnocai database, southern Ontario has a total organic carbon content of 0.27 Pg C, a value much smaller than our estimate of ~ 1.3 Pg C (Table 4). The reason that our estimated value of carbon is significantly higher than the value indicated in the Tarnocai database has to do with differences in spatial extent of different wetland types. In other words, the Tarnocai database uses similar range of carbon mass per area (171.7 ± 170.9 kg m^−2^, mean ± 1 SD, not specified for peatland types) as we use in our study (Table 4) indicating the difference has to do with how much more land is covered by different wetland types. Marsh and swamp in our estimates were one tenth the spatial extent indicated in the Tarnocai database, while fen and bog in the Tarnocai database were 8.3 and 3.4 times greater than our estimates, respectively.

Future research refining the extent of swamps and marshes, as well as their peat accumulating abilities, will help to decrease discrepancies between different carbon accounting databases. For example, in the Tarnocai database [41] swamp-rich regions like southern Ontario might be underrepresented for post-glacial peat accumulation and the Holocene wetland carbon sink. Future research should focus on using detailed substrate profiles to identify ‘past peatland conditions’ from apparently non-peat forming wetlands. Because often peat is not floating at the surface in swamps and marshes, these wetland types are not fully accounted for in the carbon balance database. Sediment coring is needed to determine if peat is in fact held in below-surface sediments (e.g., [18, 22, 42]). Our study which uses the SEO wetland peat cores to distinguish different carbon storage potentials by wetland class highlights the likeliness that total Holocene peat carbon accumulation has been underestimated in previous studies across this region. We extend this statement to include all middle latitude (temperate) regions in the Northern Hemisphere as we believe swamp and marsh sites have not been fully considered in published global-scale syntheses (e.g., [3, 5, 6, 11]).

How can we increase our knowledge of the extent and peat accumulating potentials of swamps and marshes in the past? Paleoecological studies may provide some insight into long-term carbon accumulation in swamp and marsh peats (i.e., see Fig. 1 for locations). Some Great Lake coastal marshes exhibit 1–2 m of organic-rich sediment layers (or peat) found between strata of mineral sediments, indicative of high energy environments [22, 43]. Bunting et al. [20] investigated Oil Well Bog in Ontario with shallow peat cores (~ 30–50 cm) for late Holocene wetland history; the study site where the authors suggest “the official name is misleading” as according to the vegetation found here, the site would be better classified as a swamp than a bog. Yu et al. [18] report on a peat core from a hardwood swamp near Rice Lake, Ontario. The sediment profile exhibited a peat layer ~ 2 m in thickness, but the top 50 cm was a silt-dominant mineral layer; the swamp is not currently accumulating peat but did so in the past.

Ott and Chimner [13] examined some coniferous swamp sites in Michigan and Minnesota (USA) and discovered significant woody peat accumulation in situ. These systems have not been included in the data synthesis for northern high-latitude peatlands like Loisel et al. [11]. In northern Ontario, the vast pristine forest was studied for its carbon stock behavior in the past and under future climate projections [38]; that study noted that belowground carbon was conservatively estimated as peaty forest soils have not been fully accounted. The treed-wetlands within the forest (~ 30% of the study area of [38]) need to be studied for peat deposits for Holocene long-term carbon sink potential like the extensive fen-bog peatlands dominant in more northern latitudes [44]. A preliminary examination for peat-rich sediments may help locate overlooked peat-forming swamps from extensive forested areas. For example, Beamish [45] used penetration of airborne gamma radiation to distinguish forest peat deposits from mineral soils.

## Conclusions

While the current study examined a small region, the approach presented here can be applied to other regions to better quantify the role of global wetlands in Holocene land carbon history. To fully consider the potential for natural wetlands to contribute to past land carbon sinks, our study shows that current surface conditions cannot be used to estimate past wetland carbon stocks. Also, many forested wetlands and marshes are likely to be underestimated in regional carbon inventories as they are often considered coeval with upland forest systems or mineral soil wetlands, with below ground carbon stocks considered much less significant than typical peatlands (bogs and fens).

Temperate wetlands have experienced high anthropogenic pressures, prior to efforts to document their important contribution to land carbon sequestration. Proper assessment of the converted wetlands can help recognize the overlooked portion of Holocene peatland carbon sink. Mid-latitude peat deposits have possibly acted as significant land carbon sink under natural conditions but have been recently transformed into carbon sources by wetland drainage and anthropogenic land use. There also remains the potential for on-going release of old wetland carbon as underlying peat continues to oxidize and wetlands continue to be degraded.

## Additional file

**Additional file 1: Table S1.** Comparison of wetland classification of two data sources. **Figure S1.** Wetland mapping methodology used by Snell [17] in the main text. **Figure S2.** SEO peat survey map example. **Appendix S1.** Wetland carbon stock estimation and limitation. **Appendix S2.** Botanical peat composition of the SEO peatlands. **Table S2.** Average botanical compositions of the SEO peat samples.

## Abbreviations

SEO: southeastern Ontario
ELC: ecological land classification
DUC: Ducks Unlimited Canada
SOLRIS: Southern Ontario Land Resource Information System
