# Supplementary material for: Potential carbon loss associated with post-settlement wetland conversion in southern Ontario, Canada
Source: Carbon Balance Manag. 2018 Apr 20;13:6. doi: 10.1186/s13021-018-0094-4 (PMC5910441; doi:10.1186/s13021-018-0094-4)
Supplement: Supplementary file 1 — Additional file 1: Table S1. Comparison of wetland classification of two data sources. Figure S1. Wetland mapping methodology used by Snell [17] in the main text. Figure S2. SEO peat survey map example. Appendix S1. Wetland carbon stock estimation and limitation. Appendix S2. Botanical peat composition of the SEO peatlands. Table S2. Average botanical compositions of the SEO peat samples. [file 13021_2018_94_MOESM1_ESM.docx]

Additional Information

Table S1. Comparison of Wetland Classification of Two Data Sources

| This study | SOLRIS Version 2.0^a^ | SEO Peat Survey^b^ |
| --- | --- | --- |
| Wetland | Differentiated by limited vegetation growth (e.g., <60% tree cover) from ‘Forest’ class.  Shallow standing or seasonally-fluctuated water table with hydrophilic plants.  Open water class (not wetland) is defined by >2 m depth of surface water without emergent vegetation.  Peatlands accumulate >40 cm of moss peat layers; other wetlands can be underlain by either organic/peaty or mineral substrate. | Topographic lows (basins, depressions, and adjacent low slopes) with lack of drainage ways. Seasonally or tidally flooded.  Water saturated long enough to promote hydric or organic soils and hydrophilic vegetation.  Some have seasonally variable water levels from spring floods to summer droughts.  Peatlands have >40 cm of peat layers by graminoid, woody or brown moss dominated (fen, swamp), Sphagnum peat (bog), or mixture of peaty and mineral soils (marsh). |
| Tree  Swamp | Treed (wooded) wetland with >25% cover of tree species (>5 m height).  Hydrophytic trees such as white cedar (*Thuja occidentalis*) and tamarack (*Larix laricina*) define conifer swamp. Silver maple (*Acer saccharinum*) and black ash (*Fraxinus nigra*) are indicators of hardwood swamp.  Substrate is seasonally submerged, and deep accumulation of peat is unlikely.  Waters are circumneutral to moderately acidic, mineral rich, and oxygenated to some degree.  Standing to gently flowing water tables on the surface are connected to subsurface waterflow (abundant pools and channels). | Swamp is a minerotrophic wetland, either heavily wooded (>25% tree cover) or with shrub thickets (>25% tall shrubs).  Dominant tree species subclassify tree swamp into coniferous, mixed, and hardwood (deciduous) type.  Substrate mixtures (detrital mineral/organic sediments, woody peat). If Sphagnum peat accumulates, the continuous layer is <30 cm in thickness.  Lateral or groundwater input of mineral-rich water affects wetland water condition.  Hummocky surface broken by wet interstitial hollows (channels), or flat surface with spring pools. |
| Shrub  Swamp | Thicket Swamp class (<25% tree cover). Dominated by hydrophytic shrubs (>25%) such as alder (*Alnus rugosa*), dogwood (*Cornus stolonifera*), and willow (*Salix* spp.).  Other characteristics same as Tree Swamp class described above. | Dominance of tall shrubs (>1.5 m height) by >25% in cover (<25% tree cover).  Often grades into shrub-rich Marsh but distinguished by its firm, rather consolidated peat surface, less connection to open streams, and denser and taller shrub cover. |
| Fen | Minerotrophic peatland.  Open, shrub and treed types.  Tree (>2 m height) cover <=25%. Sedges, grasses and low (<2 m) shrubs dominate.  Sedge and brown moss (peat) substrate.  Alkaline to mildly acidic waters.  Rare in southern Ontario. | Open or sparsely wooded minerotrophic wetland on depressed surface, except for low hummocks or ridges often being transitional to Bog.  Dominated by sedges, grasses, and mostly non-ericaceous shrubs (tree cover <25%).  Graminoid, woody or brown moss peat. If with abundant Sphagnum moss, not underlain by its continuous peat thicker than >30 cm. |
| Bog | Ombrotrophic peatland.  Open, shrub and treed types.  Tree (>2 m height) cover <=25%.  Sphagnum peat substrate.  Acidic water conditions prevail.  Rare in southern Ontario. | Ombrotrophic or weakly minerotrophic peatlands.  Sparse graminoids and shrubs (<25% cover, <2 m tall) without tall trees (>10 m height).  Raised surface (hummock-hollow) with Sphagnum moss carpet.  Acidic water (pH <4.4) and isolated from mineral-soil water movement. |
| Marsh | Wet areas periodically or permanently inundated with standing or moving water.  Tree and shrub cover <25%. Emergent hydrophytic macrophytes dominate (rushes, reeds, grasses, and sedges). Low shrubs (sweetgale, red osier, and winterberry) may also occur.  Substrate usually consists of mineral or organic soils with a high mineral content, but there may be as much as 2 m of peat accumulation.  Usually circumneutral to slightly alkaline water with relatively high oxygen level. Water remains within the plant root zone during at least part of the growing season. | Well-defined aquatic basins or shoreline zones transitional to deep-water areas.  Submergent, floating or emergent species in standing water <2 m deep, or on seasonally exposed substrate. Sedges, grasses, cattails, and reeds (>25% cover), or low shrubs (<25% cover).  Inorganic or organic substrates exposed to wave or water flow. Peat may accumulate in less disturbed sites (e.g., less exposed to erosion).  Periodically or permanently flooded by mineral and nutrient rich water from lake, river, or groundwater flow. Season or tide induced drought may happen. Often graded into Shrub Swamp. |

^a^Land classification of SOLRIS (Southern Ontario Land Resource Information System) is based on Ecological Land Classification (ELC) of Ontario. The new release (SOLRIS Version 2.0) performed differentiation of ‘thicket’ and ‘treed’ swamp, improvement from monotonous swamp class in the previous version [1]. This table contains some direct quotes from the data specification of SOLRIS metadata.

^b^Wetland classification for the SEO (southeastern Ontario) peatland survey was accomplished by cumulative research on Canadian wetlands in various locations [2]. This table summarizes the classification performed for the original mapping in the survey.

Figure S1. Wetland mapping methodology used by Snell [17] in the main text


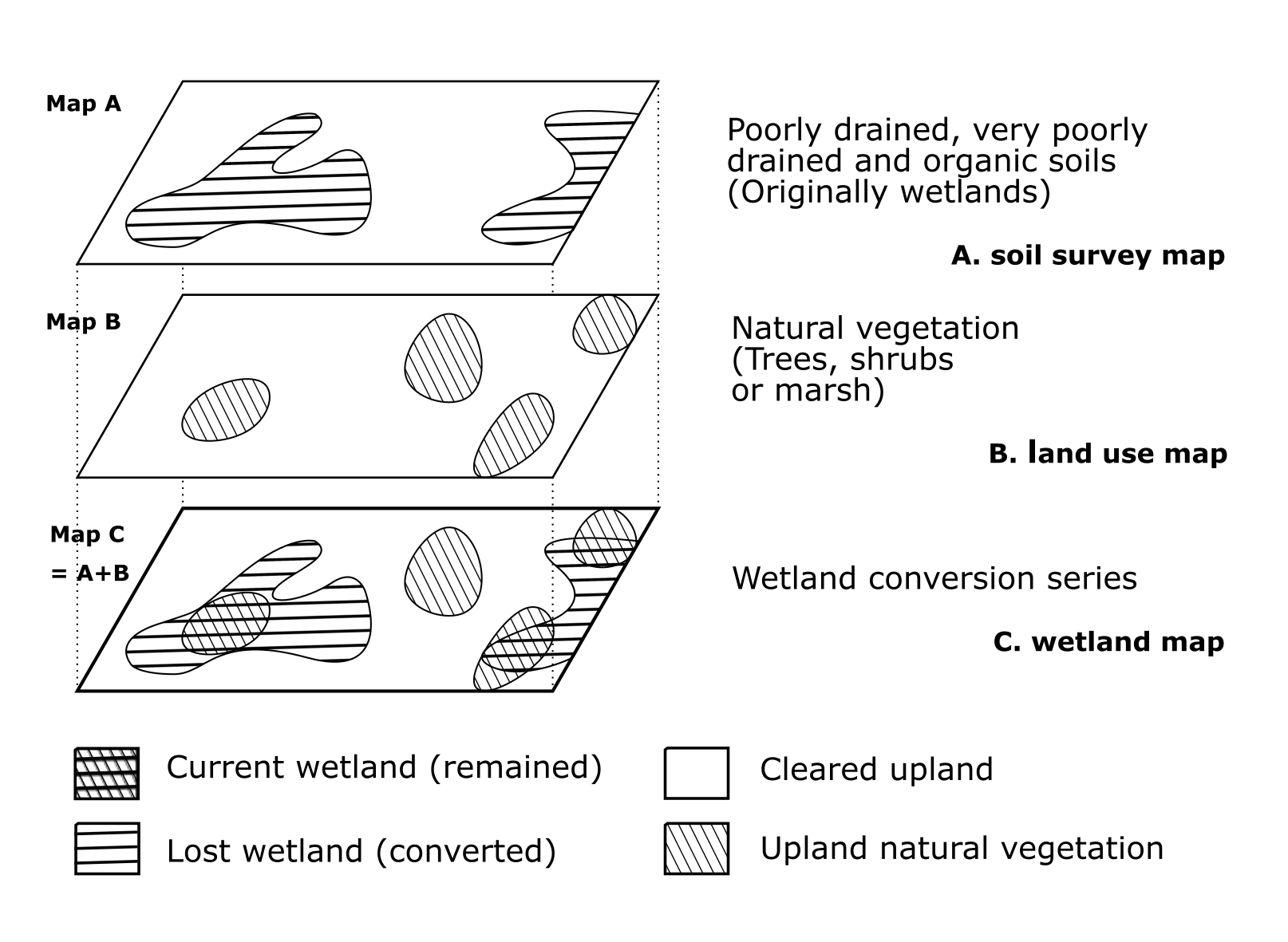


Modified from Figure 1 of *An evaluation of a methodology (Snell, 1981)*

*for determining presettlement and existing wetlands in Canada*

by Ingrid Kessel-Taylor, 1983, Environment Canada, Final Report.

Original figure legend:
“Figure 1: Wetland Mapping Methodology Using Existing Soils and Vegetation Mapping. Source: Snell, 1981.”

Figure S2. SEO peat survey map example


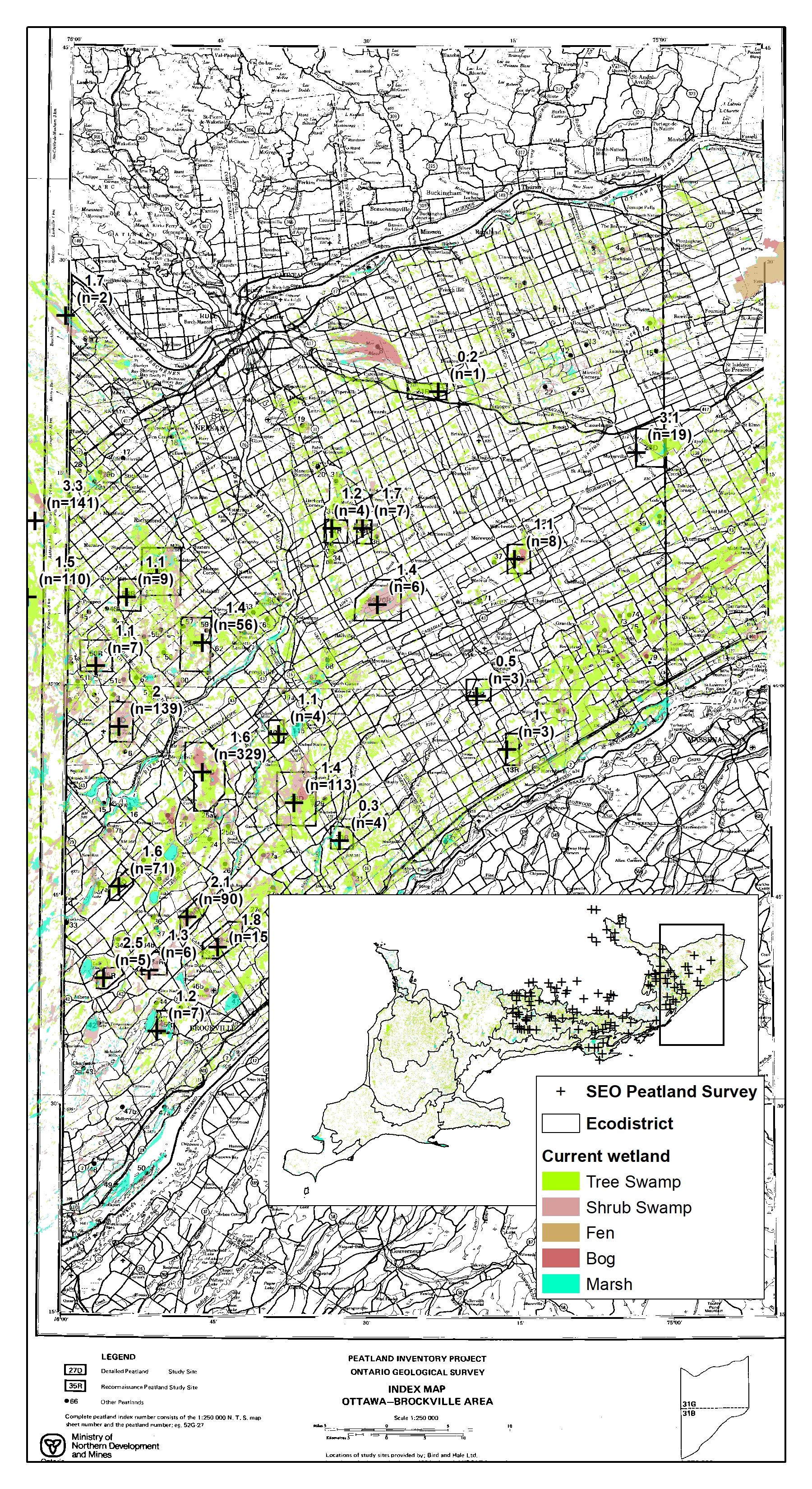


This figure shows one region of the SEO peatland survey report. The wetland classes are same as presented in Figure 2 in the main text. The labels show mean peat depths of the surveyed sites with the number of peat cores in the parentheses. The inset map corresponds to Figure 1 of the main text with peat depth measurement points.

Appendix S1. Wetland Carbon Stock Estimation and Limitation

Average Peat Depths by Wetland Types

The SEO peatland survey was aimed (1982-1984) to evaluate the potential economic benefits from peat extraction [2]. Total peat volume was determined by extensive peatland mapping (air photo interpretation supported by field investigation) with the average peat depths (Table 2) for the resource survey. Peat depths were recorded for all peat cores and the surface peatland type classified as marsh, bog, swamp, and fen (main formations) with subformations (open or treed) based on the survey’s classification system (Table S1). Marsh and swamp classes described in the peatland survey are compatible with the current wetland classification system (Table S1) and show peat depths exceeding 40 cm, comparable to the wetland sediment cores used for paleoecological in southwestern Ontario marsh and swamp sites (Figure 1 in the main text for locations and references). Therefore, we assume for the southern Ontario study area that all wetlands potentially have the same average peat depths obtained from the SEO peatland survey dataset (Table 2 of main text).

Peat Organic Carbon Content and Density

Some peat cores in the survey were analyzed in the laboratory for detailed physical and chemical compositions [2]. Total carbon content (C-TTL, %) was measured by a combustion method, and organic carbon content (C-Org, %) was by the same process after the acid treatment [3]. Southern Ontario wetlands have developed on the carbonaceous substrates (limestone bedrock eroded by glacial activity), therefore it is possible that inorganic carbon is non-negligible in these cores, contrary to what is often assumed. Organic carbon measurements were not available for all the peat cores in the survey dataset. Ash content (Ash, %) was available for almost all the samples, determined by ignition loss in a muffle furnace with sufficient oxygen (at 750°C for 1 hour). This measurement is often used for deriving organic matter (OM) content (OM% = 100 – Ash%) [2,3]. To measure organic matter content, the ignition temperature is usually set for 550°C (loss-on-ignition; LOI), and the organic carbon percentage is approximated by applying a conversion factor 0.5-0.55 to the organic matter percentage [4-6]. Given that the higher temperature setting of the survey’s method could partially burn the inorganic carbon [7], we applied a new conversion factor adjusted for the survey’s dataset. From the data points having both Ash (%) and C-Org (%) values, the mean of C-Org/(100-Ash%) was calculated as 0.47 (SE=0.008, n=209) and used for converting OM% (derived from Ash%) to organic carbon percentage (OC% = OM% × 0.47) for this study’s calculation.

The organic carbon density was estimated from dry bulk density and the OC% (i.e., organic carbon density [kg C m^-3^] = dry bulk density [g cm^-3^] × OC%). The organic carbon densities of 287 sample points were categorized into each peatland type for mean and error calculations (Table 3). The same procedures were performed without categorizing the surface types giving a single representative value for the carbon density (‘All Samples’ in Table 3 of the main article).

Cumulative Carbon Mass and Wetland Carbon Stock Estimation

The cumulative carbon mass (kg C m^-2^) was determined for each wetland class by the product of the average peat depth (Table 2) and organic carbon density (Table 3) (i.e., cumulative carbon mass [kg C m^-2^] = organic carbon density [kg C m^-3^] × peat depth [m]) (Table 4). Then, potential carbon storage for the pre-settlement and current wetlands were quantified by the sum of each wetland class (Table 4). Before this step, the pre-settlement wetlands found to be currently classified as cliffs, beach (open sand deposits along the coast), or alvars (exposed bedrock, with or without vegetation) were excluded from the area calculation because such areas are unlikely to have had any significant peat layers.

There was no subdivision for the tree swamp class of the study’s wetland maps based on SOLRIS V2.0. Therefore we used the SOLRIS land classification of ‘forest’ to portion treed swamps according to the same ratio for different type of forest (coniferous : mixed : deciduous = 22 : 25 : 54), speculating the similar level of tree dominance between ‘forests’ and ‘forested wetlands.’ This process was performed to not overestimate the average peat depth for the tree swamp class, given that the conifer swamps often have significantly deeper peat layers than the other two swamp groups [2]. Overall a lower frequency of coniferous swamp was expected for southern Ontario because of the contribution of the southwestern ‘Ecoregion 7E’ with more deciduous tree cover than conifer (see details below).

Statistics for the Tree Swamp Peat Depths and Carbon Mass

The SEO peatland classification was analogous to SOLRIS for the four wetland types of thicket (= shrub) swamp, fen, bog, and marsh (Table S1), but the three subgroups of the treed swamp were not available by SOLRIS V2.0 as the treed swamp category did specifydominant species. Statistical analysis was conducted for the swamp peat depths (Table 2 in the main text) from the SEO survey using R statistics 3.3.3. The result of ANOVA (Analysis of Variance) showed significant variation among the conifer, mixed, and deciduous (= hardwood) swamp groups as F(2, 74) = 4.828 and p = 0.0107. Tukey’s HSD test for multiple comparisons indicated a statistically significant difference in the average peat depths of conifer and deciduous swamps (p = .0075) but not between conifer and mixed (p=0.1727) in 95% confidence level.

Similarly, the three swamp subgroups have different mean organic carbon densities (Table 3 in the main text), and the statistical analysis also noted significant variation among the three groups, F (2, 160) = 15.26, p < 0.001 by ANOVA. The Tukey method suggested a difference between mixed and conifer swamps (p < 0.001), between mixed and deciduous swamps (p = 0.033), but not between conifer and deciduous (p = 0.171) in 95% confidence level. Such differences affected the estimation of the cumulative carbon mass as well. Specifically, the coniferous swamp has almost double the carbon mass than the deciduous swamp (Table 4 in the main article). Therefore, instead of averaging all the carbon quantities from the three different swamps (conifer, mixed, and deciduous; to give one representation for the single ‘tree swamp’ cover), each value was applied respectively by partitioning the tree swamp extent into three types in proportion to the SOLRIS forest cover. Then, the three resulting carbon values were summed to give the estimate for the total tree swamp carbon stock (Table 4).

Limitations

The uncertainties for the carbon stock values could be greater than the ranges presented in Table 4. First, we were not able to present errors for the wetland maps and area calculations. At least for the current wetland map, there is an accuracy assessment report for the previous version of the same land survey (SOLRIS V1.2), which might apply to the new version as they used the same procedure to delineate wetlands. While not all wetland types were considered, swamp and marsh classes were examined for classification errors [8]. For both wetlands, the map shows ~80% accuracy for location and extent (the complete overlap with reference wetlands). If we do not value the exact boundary matching (locations), which is less important for giving the total carbon stock potential, the wetland map showed almost the same abundance of wetlands (96.6% match by total extent). We assumed that the current wetland extent was reliable for calculating the carbon storage (Table 5). For the past wetlands, there was no available reference to assess the accuracy of resulting map, other than accepting the methodology of the original studies, or comparision with other methodologies for predicting potential wetland extent. The total mapped pre-settlement wetlands in our study was 30% of the total study area, which might show the full potential for naturally available wetland cover. For comparison, Fan et al. [9] proposed shallow water table depth (< 2 m) measured and simulated from local hydrologic conditions as an indicator of waterlogged terrain, and the 30% cover is near the upper limit of the simulated land cover range (22 to 32%) globally.

Categorization of the pre-settlement wetlands is also uncertain with regard to our reliance on the current wetland distribution. Nevertheless, different cumulative carbon masses for categorized wetlands were attempted rather than using the single representative value (which had been done in previous studies) for total wetland extent (Table 4). The difference seemed not considerable but will become much more significant at the global scale, which would incorporate a broader range of peat types and wetlands.

Also, we acknowledge the lack of peat core data for southwestern Ontario and underrepresented uncertainty of carbon stock estimation in terms of peat depth and carbon density values. It is a fundamental problem for carbon stock assessment. Especially, peat depth likely influences a major part of the uncertainty given the larger variation among the sites, also recognizable from the SEO dataset we used. According to a comprehensive calculation by Loisel et al. [11], both carbon content (%) and bulk density of individual peat samples can be assumed within certain ranges depending on peat types, and the scale of variation is much less than that of peat depths by peatland locations. Beilman et al. [4] suggested the use of mean peat depth might reasonably quantify a peatland carbon stock in regional scale (tens of thousands square kilometers) by averaging out the variability, while prediction of peat depth in finer scale (less than hundreds square kilometers) was more challenging with higher uncertainties.

Appendix S2. Botanical Peat Composition of the SEO Peatlands

In the SEO peatland field survey, peat cores were examined for changing “botanical composition” (wood, sedge, and moss peat) from top to bottom [3]. Any noticeable changes in peat composition was used to infer vertical structure and history of the peatland site; also, these data could be used to define the intervals of the longer peat cores and contribute to deciding the subsampling points for further physical and chemical analysis in the laboratory [2]. The relative abundance of each botanical type among ‘Moss’ (S), ‘Sedge’ (C), and ‘Wood’ (L) was scored on a scale of 0 to 10, expressing roughly the percentage compositions (0-100%) of peat material. The scores were averaged giving the general view of the different peat composition for each peatland class.

Table S2 summarizes the relative peat compositions of the seven peatland classes with apparent botanical peat types among moss, sedge, and wood. In the left half, the averages represent down to the full depths of the examined peat cores. The proportion of the moss-like peat is highest below the bog cover (there are no moss observations from the marsh samples). The moss residues rarely contribute the peat accumulation under the deciduous swamp but do to some degree for the conifer and shrub swamp peat layers (~10-20%). The wood peat is dominant for any swamp peat layers, especially in deciduous, but also found to be abundant under some fen sites as well. The herbaceous peat comprised nearly half of the peat layers overall but was particularly plentiful in the marsh sediments. When only the top intervals (surface to about 30 cm in depth) of the peat core sections were considered (the right half of Table S2), variations in the peat botanical compositions became more straightforward with regard to the different peatland covers. For example, the near surface part of the bog cores is almost full of the moss peats, while the deciduous swamp peat layers mostly consist of the wood-derived peat. Altogether, for the near surface there is less contribution of the sedge peat at the expense of more wood and moss peats compared to the full depth averages (see the bottom row of Table S2).

Although the botanical composition of the older peat layers may lead to a biased conclusion for the actual vegetation cover at the time [10], overall increase of wood peat to the shallow depths may support a tendency to have more tree species over time (relative increase of wood peat counts and decrease of sedge at the top interval compared to the full depth average, Table S2).

For the bog and fen peatlands, the transition of the surface cover from sedge and other moss dominance (rich fen) to *Sphagnum* colonization (poor fen and bog) is common for the northern peat profiles [11]. In Table S2, the clear dominance of moss peat only at the top interval of bog cores is attributable to the fen-bog transition observed from many northern peatlands during the Holocene [11].

Table S2. Average Botanical Compositions of the SEO Peat Samples

|  | Number of All Sections | Observed Peat Type^a^ | | |  | Number of Top Sections | Observed Peat Type | | |
| --- | --- | --- | --- | --- | --- | --- | --- | --- | --- |
|  |  | Moss | Sedge | Wood |  |  | Moss | Sedge | Wood |
| Conifer Swamp | 88 | 1.1  (2.6) | 4.0  (4.1) | 5.0  (4.1) |  | 17 | 3.2  (3.8) | 1.7  (2.5) | 5.1  (3.9) |
| Mixed Swamp | 47 | 0.6  (2.0) | 5.0  (3.8) | 4.4  (3.9) |  | 12 | 1.4  (3.0) | 3.1  (2.5) | 5.7  (3.7) |
| Deciduous Swamp | 25 | 0.04  (0.2) | 3.6  (4.2) | 6.3  (4.2) |  | 8 | - | 0.1  (0.4) | 9.9  (0.4) |
| Shrub Swamp | 34 | 1.5  (3.2) | 5.3  (4.1) | 3.2  (3.6) |  | 9 | 2.1  (3.3) | 2.4  (2.8) | 5.4  (3.9) |
| Fen | 37 | 0.5  (1.7) | 5.5  (3.6) | 3.9  (3.6) |  | 10 | 1.4  (3.0) | 4.1  (3.2) | 4.5  (3.5) |
| Bog | 45 | 5.1  (4.1) | 3.6  (3.8) | 1.3  (1.8) |  | 9 | 8.6  (1.8) | 0.7  (1.3) | 0.8  (1.2) |
| Marsh | 9 | - | 8.8  (1.1) | 1.2  (1.1) |  | 2 | - | 8.0  (0.0) | 2.0  (0.0) |
| All  (95% CI)^b^ | 285 | 1.5  (1.1-1.9) | 4.6  (4.1-5.0) | 3.9  (3.5-4.4) |  | 66 | 2.7  (1.8-3.7) | 2.3  (1.6-2.9) | 5.0  (4.1-6.0) |

Mean and standard deviation (SD) calculated for each peatland class with the peat samples fully recognized for the three peat types, excluding the intervals with values of ‘other’ peat type [2]. Full sections count all intervals of the peat cores from top to bottom. Average peat compositions for the top sections (the first top interval of the peat cores, unless the bottom depths > 1 m; 0.33 m on average) are summarized as Mean and SD on the right side of the above table.

^a^Determined by visual interpretation of retrieved peat; data are given using a 1 to 10 scale as the approximate percentage of each peat type’s relative composition to the whole identifiable peat material [3].

^b^Lower and upper limit of 95% confidence interval (CI) for the mean, i.e., Mean ± 1.96*SEM

References

1. SOLRIS Technical Team. Southern Ontario Land Resource Information System (SOLRIS) Version 2.0: Data Specifications. 2015.

2. Riley JL. Peat and peatland resources of southeastern Ontario. Ontario Geological Survey. Miscellaneous Paper 154. 1994. p. 167.

3. Riley JL. Laboratory methods for testing peat—Ontario peatland inventory project. Ontario Geological Survey. Micellaneous Paper 145. 1989. p. 51.

4. Beilman DW, Vitt DH, Bhatti JS, Forest S. Peat carbon stocks in the southern Mackenzie River Basin: uncertainties revealed in a high-resolution case study. Glob Chang Biol. 2008;14:1221-32.

5. Gorham E. Northern peatlands: role in the carbon cycle and probable responses to climatic warming. Ecol Appl. 1991;1:182-95.

6. Heiri O, Lotter AF, Lemcke G. Loss on Ignition as a Method for Estimating Organic and Carbonate Content in Sediments : Reproducibility and Comparability of Results. J Paleolimnol. 2001;25:101-10.

7. Wang Q, Li Y, Wang Y. Optimizing the weight loss-on-ignition methodology to quantify organic and carbonate carbon of sediments from diverse sources. Environ Monit Assess. 2011;174:241-57.

8. SOLRIS Technical Team. Accuracy assessment report 2: SOLRIS Version 1.2 (April 2008 release). Peterborough, Ontario; 2008.

9. Fan Y, Li H, Miguez-Macho G. Global Patterns of Groundwater Table Depth. Science. 2013;339:940–3.

10. Clymo RS. The Limits to Peat Bog Growth. Philos Trans R Soc B Biol Sci. 1984;303:605–54.

11. Loisel J, Yu Z, Beilman DW, Camill P, Alm J, Amesbury MJ, et al. A database and synthesis of northern peatland soil properties and Holocene carbon and nitrogen accumulation. The Holocene. 2014;24:1028–42.
